# Application of Near-infrared Spectroscopy and Multiple Spectral Algorithms to Explore the Effect of Soil Particle Sizes on Soil Nitrogen Detection

**DOI:** 10.3390/molecules24132486

**Published:** 2019-07-07

**Authors:** Shupei Xiao, Yong He

**Affiliations:** 1 College of Biosystems Engineering and Food Science, Zhejiang University, Hangzhou 310058, China; 2 Key Laboratory of Spectroscopy Sensing, Ministry of Agriculture, Hangzhou 310058, China

**Keywords:** soil nitrogen, near-infrared spectroscopy, soil particle sizes, multiple spectral algorithms, mixing spectra

## Abstract

Soil nitrogen is the key parameter supporting plant growth and development; it is also the material basis of plant growth. An accurate grasp of soil nitrogen information is the premise of scientific fertilization in precision agriculture, where near-infrared (NIR) spectroscopy is widely used for rapid detection of soil nutrients. In this study, the variation law of soil NIR reflectivity spectra with soil particle sizes was studied. Moreover, in order to precisely study the effect of particle size on soil nitrogen detection by NIR, four different spectra preprocessing methods and five different chemometric modeling methods were used to analyze the soil NIR spectra. The results showed that the smaller the soil particle sizes, the stronger the soil NIR reflectivity spectra. Besides, when the soil particle sizes ranged 0.18–0.28 mm, the soil nitrogen prediction accuracy was the best based on the partial least squares (PLS) model with the highest Rp^2^ of 0.983, the residual predictive deviation (RPD) of 6.706. The detection accuracy was not ideal when the soil particle sizes were too big (1–2 mm) or too small (0–0.18 mm). In addition, the relationship between the mixing spectra of six different soil particle sizes and the soil nitrogen detection accuracy was studied. It was indicated that the larger the gap between soil particle sizes, the worse the accuracy of soil nitrogen detection. In conclusion, soil nitrogen detection precision was affected by soil particle sizes to a large extent. It is of great significance to optimize the pre-treatments of soil samples to realize rapid and accurate detection by NIR spectroscopy.

## 1. Introduction

As the main source of nutrient supply for plant growth, the nutritional status of soil is one of the key factors directly influencing plant growth and development [1]. During the plant growth process, plants obtain available nitrogen through decomposition of organic nitrogen and subsequent nitrogen mineralization (ammonification and nitrification) by microbes [2]. Thus, it is of great importance to obtain soil nutrient content such as soil nitrogen quickly and accurately for precision fertilization and agricultural production [3]. The traditional chemical method for detecting soil nitrogen content, such as Dumas combustion [4], achieves high accuracy. However, the whole detection process is complex and time-consuming [5]. At present, near-infrared (NIR) spectroscopy has been successfully applied in the fields of agriculture, food, medicine, petroleum and chemistry [6], and many scholars have applied NIR spectroscopy to detect soil nitrogen content as well.

Firstly, NIR spectroscopy could be used as a rapid, inexpensive and non-destructive technique to predict the physical, chemical and biochemical properties of soil. The predicted results were evaluated as excellent (R^2^ > 0.90) for soil organic carbon, Kjeldahl nitrogen, soil moisture, cation exchange capacity, microbial biomass carbon, basal soil respiration, acid phosphatase activity and β-glucosidase activity [7]. Soil nitrogen was detected with multiple linear regression (MLR) method at the spectral bands of 1702, 1870 and 2052 nm using NIR spectroscopy [8], and the correlation coefficients between measured and predicted values of soil nitrogen achieved 0.931 [9]. Moreover, it was found that the sensitive bands of soil total nitrogen were different for different soil types and the characteristic bands were affected not only by soil type, but also by sampling depth [10]. Secondly, the effect of soil particle size on soil nitrogen detection by NIR spectroscopy was also studied. Hernandez et al. found that the NIR prediction result of soil organic nitrogen was not ideal when the soil particle size was too large or too small [11]. On this basis, scholars have carried out further studies. When soil particle sizes were in the range of 0.5–5 mm, the correlation coefficient of prediction was higher than 0.8; the prediction accuracy was worse when the soil particle size was less than 0.25 mm or greater than 0.5 mm [9]. However, there were different conclusions about the effect of soil particle sizes on soil nitrogen detection using NIR spectroscopy. For example, in Cozzolino’s research, in which the correlation coefficients of coarse sand (0.25–2 mm), fine sand (0.05–0.25 mm) and clay sand (<0.05 mm) were 0.90, 0.92 and 0.96, respectively, between soil nitrogen and NIR spectra [12]. Similar to Cozzolino’s research, Zhu et al. pointed out that the smaller the soil water content and soil particle size, the better the prediction accuracy [13]. Furthermore, the results of Nie’s research suggested that the soil with the strictest pretreatments (dried, ground, sieved and pressed) achieved the highest accuracy in predicting the soil nitrogen content using NIR sensor [14]. 

It is concluded that the accuracy of detecting soil nitrogen content is largely affected by soil particle size. However, at present, the research on the influence of soil particle sizes on soil nitrogen detection by NIR mainly has the following shortcomings: First, soil particle size classification is not specific enough and lacks systematic research; second, qualitative or quantitative analysis of the mixed spectra based on different soil particle sizes is lacking; third, the modeling method for unified data is relatively simple, and data stability needs further study. In order to solve the above problems, the main objective of this study was to (1) systematically study the effect of soil particle sizes on the detection of soil nitrogen by NIR spectroscopy; (2) conduct a qualitative and quantitative analysis of mixed spectra based on different soil particle sizes; (3) model and analyze the soil NIR spectra by four spectral pretreatment methods and five modeling methods, attempting to achieve high feasibility and reliability.

## 2. Results and Discussion

### 2.1. Analysis of Soil NIR Spectrum

**Figure 1:**
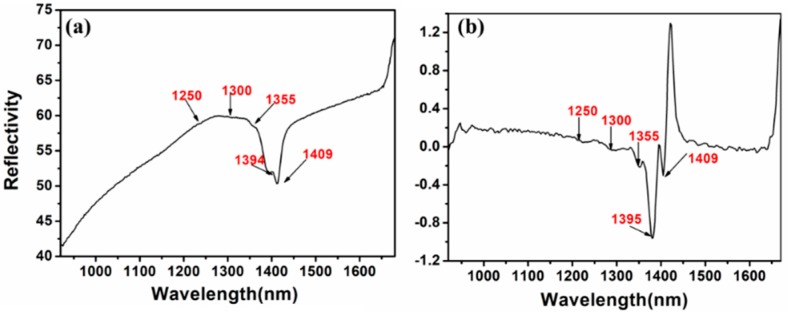
The near-infrared (NIR) spectrum of soil: (**a**) original spectrum; (**b**) first-order spectrum.

The soil type, color, and other physicochemical properties will affect the spectral characteristics of soil to a large extent. Therefore, before exploring the effect of soil particle sizes on soil nitrogen detection using NIR spectroscopy, the spectral properties of the red soil used in this experiment were analyzed. The original NIR spectrum of soil is given in Figure 1a and the NIR spectrum of soil with first-order pretreatment is shown in Figure 1b.

According to Figure 1, there were strong absorption peaks at 1394 and 1409 nm, which belonged to the hydrogen group vibrations of N-H band and O-H band [15]. Furthermore, it can be seen that the absorption peaks at 1250, 1300 and 1355 nm had weak vibrations. To be more specific, the absorption peaks at 1250 nm were assigned to the vibrations of the C-H band and the absorption peaks at 1300 and 1355 nm were assigned to the vibrations of the N-H band [16]. To a certain extent, it was indicated that the reflectivity of soil NIR spectrum could reflect the soil nitrogen level through some certain bands.

### 2.2. Soil NIR Spectra with Different Soil Particle Sizes

**Figure 2:**
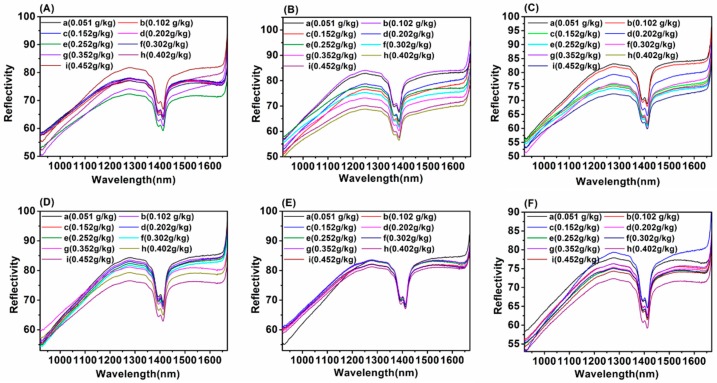
The average spectral reflectivity of soil with different soil particle sizes: (**A**) 1–2 mm; (**B**) 0.45–1 mm; (**C**) 0.28–0.45 mm; (**D**) 0.18–0.28 mm; (**E**) 0–0.18 mm; (**F**) 0–2 mm.

**Figure 3:**
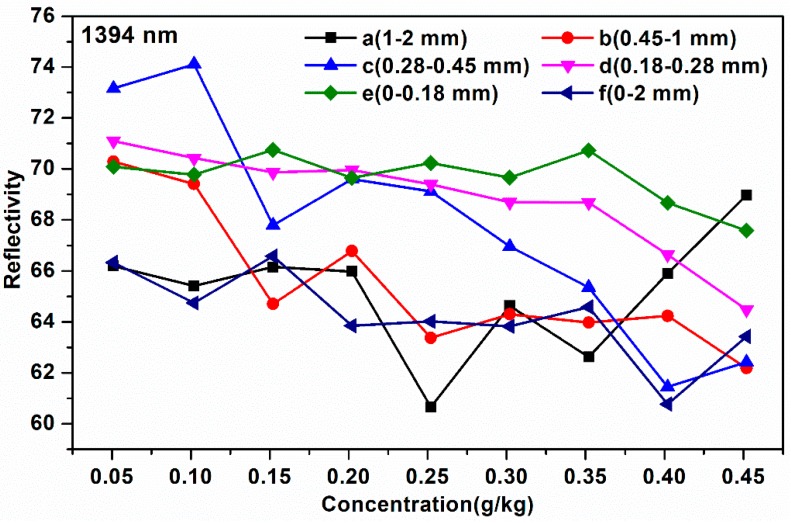
The average spectral reflectivity of soil with different soil particle sizes at 1394 nm: (**a**) 1–2 mm; (**b**) 0.45–1 mm; (**c**) 0.28–0.45 mm; (**d**) 0.18–0.28 mm; (**e**) 0–0.18 mm; (**f**) 0–2 mm.

In this paper, the average reflectivity of soil NIR spectra with different particle sizes was collected and the corresponding reflectivity curves are presented in Figure 2. The average spectral reflectivity of soil with different soil particle sizes at 1394 nm is shown in Figure 3.

According to Figure 2, on the one hand, from the perspective of the relationship between soil particle sizes and soil NIR spectra, when the soil particle sizes were 1–2 mm and 0–2 mm (Figure 2A,F), the soil reflectivity curves with different nitrogen contents were hard to separate, especially at 1394 nm (Figure 3a, f). The reason for this might be that the large soil particle sizes caused the surface of soil tablet to be uneven. Besides, when the soil particle sizes were in the range of 0.18-0.45 mm (Figure 2B–D), the spectral curve was evenly distributed. The soil reflectivity curves were concentrated when the soil particle sizes were the smallest (Figure 2E), which indicated that the smaller the soil particle sizes, the smaller the impact of soil particle sizes on the NIR reflectivity intensity. A good explanation for this is that the small soil particle sizes led to the smooth soil surface, thus resulting in the concentrated soil spectral intensity [17]. On the other hand, from the perspective of the relationship between soil NIR spectra and soil nitrogen contents, it can be seen that with the increase of soil nitrogen contents from 0.051 to 0.452 g/kg, the soil average spectral reflectivity decreased gradually (Figure 2 and Figure 3), especially for the soil NIR spectra with the soil particle sizes in the range of 0.18–0.28 mm at 1394 nm (Figure 3d). The reason for this might be that when the soil nitrogen contents increased, the absorption of soil nitrogen increased correspondingly.

### 2.3. Model Analysis of Spectral Data with Different Soil Particle Sizes

**Figure 4:**
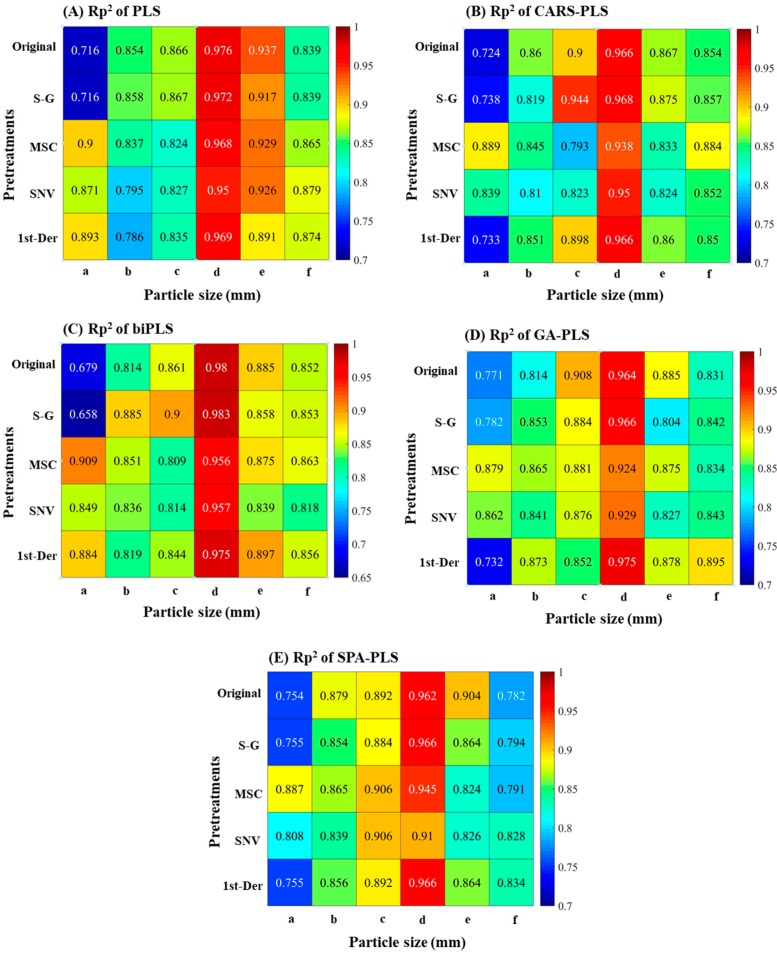
The prediction determination coefficients of four different preprocessing methods and five different models. Models: (**A**) partial least squares (PLS); (**B**) competitive adaptive reweighted sampling—partial least squares (CARS-PLS); (**C**) backward interval partial least squares (biPLS); (**D**) genetic algorithm—partial least squares (GA-PLS); (**E**) successive projections algorithm—partial least squares (SPA-PLS). Soil particle sizes: (a) 1–2 mm; (b) 0.45–1 mm; (c) 0.28–0.45 mm; (d) 0.18–0.28 mm; (e) 0–0.18 mm; (f) 0–2 mm.

**Figure 5:**
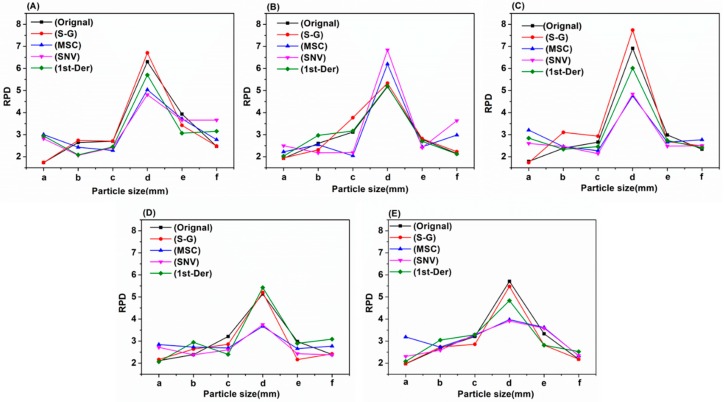
The residual predictive deviation (RPD) of four different preprocessing methods and five different models. Models: (**A**) PLS; (**B**) CARS-PLS; (**C**) biPLS; (**D**) GA-PLS; (**E**) SPA-PLS. Soil particle sizes: (a) 1–2 mm; (b) 0.45–1 mm; (c) 0.28–0.45 mm; (d) 0.18–0.28 mm; (e) 0–0.18 mm; (f) 0–2 mm.

**Figure 6:**
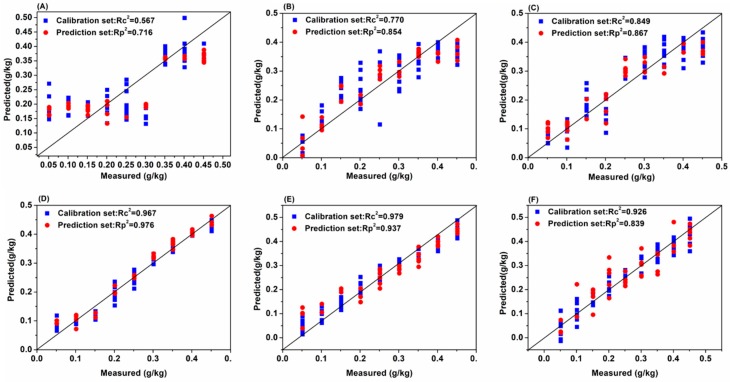
The predicted values and the measured values of the correction set and the prediction set based on PLS (original spectra). Soil particle sizes: (**A**) 1–2 mm; (**B**) 0.45–1 mm; (**C**) 0.28–0.45 mm; (**D**) 0.18–0.28 mm; (**E**) 0–0.18 mm; (**F**) 0–2 mm.

Considering the spectral information overlap and noise on the edge of acquisition band, the 930-1670 nm soil NIR spectra of six soil groups with different soil particle sizes were pretreated with Savitzky–Golay (S–G) smoothing, detrend (DT), standard normal variation (SNV), and first derivative (1st-Der), respectively, and then modeled by partial least squares (PLS), competitive adaptive reweighted sampling-partial least squares (CARS-PLS), backward interval partial least squares (biPLS), genetic algorithm-partial least squares (GA-PLS) and successive projections algorithm-partial least squares (SPA-PLS), respectively. The sample set portioning based on the joint x–y distance (SPXY) method was used to separate the soil samples into a calibration set and validation set at a ratio of 2:1 for each soil group. The modeling results of PLS with different pretreatments are presented in Table 1 and the modeling results of biPLS, CARS-PLS, SPA-PLS and GA-PLS with different pretreatments are presented in Table S1–S4, respectively. In order to compare the prediction results and the model stability of four different preprocessing methods and five different modeling method more directly, the prediction determination coefficients and residual predictive deviation (RPD) of four different preprocessing methods and five different modeling methods are given in Figure 4 and Figure 5, respectively. The scatter plot with the predicted values and the measured values of the correction and prediction sets based on PLS (original spectra) are shown in Figure 6.

As illustrated in Table 1, Table S1–S4 and Figure 4, Figure 5 and Figure 6, the conclusions were as follows:

First, from the perspective of the effect of soil particle sizes on the detection of soil nitrogen contents by NIR, the detection results were relatively poor (0.658 < Rp^2^ < 0.893) when the soil particle sizes were 1–2 mm. It was shown that large soil particle sizes were averse to soil nitrogen detection and this conclusion is consistent with Cozzolino’s and Bao’s research [9,12]. Moreover, when the soil particle sizes were in the range of 0.28–1 mm, the soil nitrogen prediction accuracy (0.45–1 mm: 0.795 < Rp^2^ < 0.885; 0.28–0.45 mm: 0.809 < Rp^2^ < 0.944) improved greatly. Additionally, when the soil particle sizes ranged 0.18–0.28 mm, soil nitrogen prediction achieved the best accuracy with the highest Rp^2^ of 0.983. However, soil particle sizes that were too small (less than 0.18 mm) were not helpful for the improvement of detection accuracy (0.824 < Rp^2^ < 0.926). Compared with the five soil groups, Rp^2^ reached 0.8–0.9 when the soil particle sizes were in the range of 0–2 mm, which indicated that the detection of soil nitrogen contents using NIR was also affected by the uniformity of soil particle sizes. Therefore, the prediction effect of six soil particle size ranges can be ranked as follows: 0.18–0.28 mm > 0–0.18 mm > 0.28–0.45 mm > 0.45–1 mm > 0–2 mm > 1–2 mm.

Second, from the perspective of the modeling results, there were some differences in the prediction effect of different models for different soil particle sizes. However, as a whole, the PLS, biPLS and CARS-PLS models performed better in terms of detection accuracy than the GA-PLS and SPA-PLS models. A possible explanation for this is that PLS performed well in summarizing the information of independent variables, which effectively handled the variables multiple correlation problem. The biPLS and CARS-PLS could efficiently select valid variables and eliminate redundant variables, which resulted in more accurate results and higher detection precision. Although GA-PLS and SPA-PLS could efficiently eliminate redundant variables to some extent, the valid information might also be incorrectly eliminated, which could lead to the relatively poor prediction results.

Third, from the perspective of spectral pretreatment methods, the soil NIR spectra processed with different pretreatment methods showed different modeling effect. Clearly, when the soil particle sizes were in the range of 1–2 mm, the prediction accuracy improved from 0.658 to 0.909 when the soil NIR spectra were processed by MSC and SNV. However, the prediction accuracy decreased when the soil particle sizes were small (0–0.28 mm). The reason could be that MSC and SNV could efficiently eliminate the influence of soil particle sizes, surface scattered light, and optical path change on NIR spectroscopy, which improved the spectral resolution, reduced the standard deviation between samples and separated the main characteristic peaks for quantitative analysis [18]. It can be seen that when the soil NIR spectra were pretreated by S–G smoothing and 1^st^-Der method, the prediction accuracy was not obviously improved, which indicated that the effect of noise in the original spectra was small. 

Finally, from the perspective of the model stability, it can be seen that the higher the Rp^2^, the better the model stability. No matter which method was used, the RPD of prediction models were more than 4 (> 3) mostly when the soil particle sizes were in the range of 0.18–0.28 mm, which satisfied the agriculture applications [19]. 

In summary, for the analysis of soil NIR spectra with different soil particle sizes, there were some differences in model prediction results using different pretreatment methods and modeling methods. However, when the soil particle sizes were 0.18–0.28 mm, soil nitrogen prediction accuracy achieved the highest Rp^2^ and RPD.

### 2.4. Spectral Analysis of Different Soil Particle Sizes

**Figure 7:**
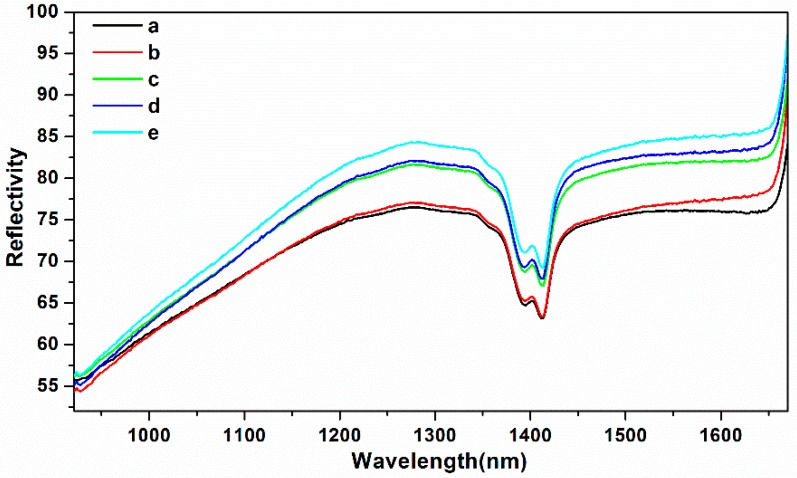
The average spectra of different soil particle sizes: (**a**) 1–2 mm; (**b**) 0.45–1 mm; (**c**) 0.28–0.45 mm; (**d**) 0.18–0.28 mm; (**e**) 0–0.18 mm.

In order to further explore the influence of soil mixing spectra with different particle sizes on soil nitrogen detection, the spectra of five different soil particle size ranges (1–2 mm; 0.45–1 mm; 0.28–0.45 mm; 0.18–0.28 mm; 0–0.18 mm) were mixed with each other in equal proportions and modeled by PLS. We obtained five average spectra (108 samples of each average spectra) of different soil particle sizes before exploring the modeling effects of different mixing spectra (Figure 7).

**Figure 8:**
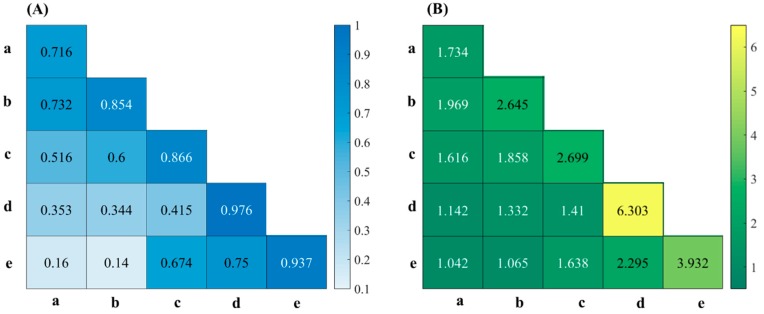
The Rp^2^ (**A**) and RPD (**B**) of PLS model with different soil particle sizes: (a) 1–2 mm; (b) 0.45–1 mm; (c) 0.28–0.45 mm; (d) 0.18–0.28 mm; (e) 0–0.18 mm.

As shown in Figure 6, with the increase of soil particle sizes, the soil NIR reflectivity curve increased correspondingly, which indicated that the bigger the soil particle sizes, the stronger the soil NIR reflectivity. The reason might be that the large soil particles caused the surface of the soil tablet to become uneven. We could infer that when the nitrogen content in soil was the same, the spectra of different soil particle sizes would also have significant differences. Therefore, we established the PLS model based on mixing spectra of different soil particle sizes with each other. The Rp^2^ and RPD of PLS model is given in Figure 8, and the PLS modeling results are presented in Table 2.

Clearly, the Rp^2^ and RPD of individual soil group were larger than those of two mixed soil groups. Moreover, the PLS modeling effect of two mixing spectra with small differences in soil particle sizes were better than those of two mixing spectra with big gap in soil particle sizes. A good explanation was that soil NIR reflectivity was affected by soil particle sizes, and the difference of soil particle sizes resulted in worse uniformity of soil groups to be detected, which would reduce the model accuracy. The results showed that the larger difference of mixing spectra with soil particle sizes, the worse the modeling accuracy. Therefore, the consistency and uniformity of soil particle size should be maintained as far as possible to achieve high accuracy when using NIR spectroscopy to detect soil nitrogen content.

## 3. Materials and Methods 

### 3.1. Experimental Materials

The experimental soil was collected from Maoming city, Guangdong province, China (N21°25′, E111°07′). The portable NIR optical instrument from Isuzu Optics Corp (Shanghai, China) is an interferometer instrument reflective with two integrated tungsten halogen lamps. This instrument collects spectral information in the range of 900–1700 nm, with an optical resolution of 10 nm. 

### 3.2. Experimental Materials and Sample Preparation

The preparation process of soil samples was as follows: First, the soil samples were dried by air and sieved with a 2 mm mesh sieve. Second, soil nitrogen concentration was detected by biuret method and Kjeldahl determination. Third, one group of soil samples with soil particle sizes in the range 0–2 mm was obtained. The other five groups of soils with different particle sizes were obtained as follows: (1) The soil samples (0–2 mm) were sieved with a 0.18 mm sieve and the soil particle sizes in the range 0–0.18 mm were obtained. (2) The remaining soil sieved through a 0.18 mm sieve was sieved again with 0.28 mm sieve and the soil particle sizes ranging 0.18–0.28 mm were obtained. (3) The remaining soil sieved through a 0.28 mm sieve was sieved again with 0.45 mm sieve and the soil particle sizes ranging 0.28–0.45 mm were obtained. (4) The remaining soil sieved through a 0.45 mm sieve was sieved again with 1 mm sieve and the soil particle sizes ranging 0.45–1 mm were obtained. (5). The remaining soil sieved through a 1 mm sieve (1–2 mm) was obtained. Thus, six soil sample groups with different particle sizes ranging (a) 1–2 mm; (b) 0.45–1 mm; (c) 0.28–0.45 mm; (d) 0.18–0.28 mm; (e) 0–0.18 mm; and (f) 0–2 mm were obtained. The urea solutions with different concentrations were mixed with the soil samples and eight gradients with different soil nitrogen concentrations were obtained (0.051–0.452 g/kg, 0.05 g/kg per gradient). Finally, the soil samples were dried by air and pressed into 10 mm × 10 mm × 2 mm blocks. There were 108 samples (12 samples for each concentration) in each group, and 648 samples in total.

### 3.3. Soil NIR Spectra Measurement

Before performing the soil NIR spectra measurement, the instrument was preheated for 15 min and prepared with blackboard and whiteboard correction operation. In order to maintain the integrity of the original soil spectra as well as achieve the rapidity during the detection process, the spectral acquisition parameter is set up as 400 points, and each spectrum is obtained by averaging three scans. When the soil spectra were measured, the samples were placed on the light source window, which avoided the phenomenon of light leakage since the size of soil sample is larger than that of light source window.

### 3.4. Data Analysis

NIR light is an electromagnetic wave between the infrared and visible light whose wavelength range is from 780 nm to 2526 nm. The spectral information originates from the vibration of the O-H, C-H and N-H groups containing hydrogen internal vibration frequency and sum frequency overlap, which can reflect the variation of organic matter in the characteristic spectral region [20]. According to the Lambert Bill absorption law [21], the spectral characteristics would change with the variation of sample composition or structure [22]. However, at the same time, it can also be affected by the soil surface texture, density and uneven distribution of internal components, such as the overlap of spectral information, the large amount of noise and the background of detected sample, which is very difficult for all redundant information of the spectral data to be eliminated [23]. Therefore, in order to achieve the purpose of qualitative or quantitative analysis of complex mixtures, it is necessary to apply chemometric methods to extract and analyze the weak chemical information in the spectral analysis [24].

### 3.5. Spectral Pretreatment Methods

In order to achieve a better model prediction effect, in this study four spectral pretreatment methods were applied to preprocess the original soil NIR spectra, that is, S-G smoothing, DT, SNV and 1st-Der. Among them, S-G smoothing is widely used to remove noise from original spectrum such as remove high frequency noise [25]. DT algorithm is mainly used to eliminate the baseline drift of the diffuse reflectance spectrum [26]. SNV algorithm can use the absorbance values of each wavelength point to satisfy a certain distribution in each spectrum, and the spectral correction was performed according to this assumption [27]. 1st-Der is able to distinguish overlapping peaks and eliminate interference from other backgrounds, which improves spectral resolution and sensitivity.

### 3.6. Spectral Modeling Methods

#### 3.6.1. Partial Least Squares

PLS is a common-used regression modeling method for analyzing spectral data based on its flexibility and reliability in dealing with the redundant spectral data [28]. During the PLS modeling process, the spectral matrix is decomposed first and the main latent variables are obtained, then the contribution rate of each latent variable is calculated. The flexibility of PLS makes it possible to establish a regression model in the case where the number of samples is less than the number of variables. In this study, the PLS model was established with the spectral data as X and the measured soil N content as Y, whose best principal factor was determined by the root mean square error of cross validation (RMSECV) [29].

#### 3.6.2. Competitive Adaptive Reweighted Sampling—Partial Least Squares Method (CARS-PLS)

CARS is a variable selection method based on the principle of “survival of the fittest” [30], which uses Monte Carlo sampling to select several samples from the calibration set for PLS modeling and repeats this process for hundreds of iterations. In the process of wavelength variable selection, CARS preserves the wavelength variable with the absolute value of PLS regression coefficient, and the wavelength invariable with small absolute value of regression coefficient is removed. In order to obtain a series of wavelength variable subsets, each subset of wavelength variables is modeled by cross validation, and the optimal wavelength variable subset is selected according to the RMSECV value [31].

#### 3.6.3. Backward Interval Partial Least Squares

BiPLS is a variable selection method based on the PLS modeling method, it aims to filter the wavelength range of PLS model and reduce the number of sub-intervals of the worst or collinear variables, which select the best principal component number according to RMSECV [32]. For selecting the best and minimum RMSECV among all the base models, biPLS is able to preliminarily locate the NIR spectral interval and rank the importance of individual intervals during the modeling process, it also can eliminate the spectral interval with poor information to obtain better modeling effect.

#### 3.6.4. Successive Projections Algorithm—Partial Least Squares (SPA-PLS)

SPA is a forward variable selection method, which uses vector projection analysis to find the variable group with minimal redundancy information to effectively eliminate the collinear, singular and instable variables in the spectra. Araujo et al. [33] first proposed the selection of spectral variables by means of SPA. Soares et al. [34] used SPA for cross-classification analysis. Since it reduces the number of variables used in the model and lowers the model complexity, the collinear between the vectors is minimized. Extracting feature wavelength modeling based on SPA-PLS has significance in actual detection because of the useful information for mining spectral data with latent variables [35].

#### 3.6.5. Genetic Algorithm—Partial Least Squares (GA-PLS)

The aim of genetic algorithm (GA) is to search for the optimal solution by simulating the natural evolution process [36]. The biggest advantage is that the global optimization search ability of GA is strong, and it is not necessary to assign the initial value to the decision variable to be optimized. The GA itself will automatically and randomly select a set of initial values from its upper and lower limits and select the global optimal solution of the parameters according to the genetic selection strategy. On this basis, GA-PLS method aims to solve the problem of multi-correlation interference and poor model fitting in the conventional regression model, and further improve the fitting and prediction accuracy of the model [26].

### 3.7. Model Evaluation Index

In this experiment, the modeling effect was evaluated by the correlation coefficient R, the root mean square error (RMSE) and the residual predictive deviation (RPD). The correlation coefficient R reflects the level of intimacy between variables, RMSE reflects the model accuracy, and RPD reflects the model prediction ability. The higher the R and RPD and the lower the RMSE, the better the prediction model performance. In this paper, R_c_ and R_p_ represent the correlation coefficient of calibration set and prediction set, respectively, and RMSEC and RMSEP represent the root mean square error of the calibration set and prediction set respectively. Besides, RPD was suggested to be at least 3 for agriculture applications; 2 < RPD < 3 indicates a model with a good prediction ability; 1.4 < RPD < 2 is an intermediate model requiring some improvement; and RPD < 1.4 indicates a poor model prediction ability [19]. In addition, all above-mentioned data analysis in this study was performed on OMNIC v8.2 (Thermo Nicolet Corp., Madison, WI, USA) and MATLAB R2018a (The MathWorks, Inc., Natick, MA, USA). 

## 4. Conclusions 

In this paper, the variation law of soil NIR spectra with soil particle sizes was studied. The results showed that the smaller the soil particle sizes, the stronger the reflectivity of soil NIR spectra. When the soil particle sizes ranged 0.18–0.28 mm, soil nitrogen prediction achieved the best accuracy based on PLS model with the highest Rp^2^ of 0.983, the RPD of 6.706. The detection accuracy was not ideal when soil particle sizes were too large (1–2 mm) or small (0–0.18 mm). In addition, the relationship between the mixing spectra of six different soil particle sizes and the soil nitrogen detection accuracy was studied. It was shown that the larger the difference of soil particle size, the worse the soil nitrogen detection accuracy. In conclusion, soil nitrogen detection precision was affected by soil particle size to a large extent. It is of great significance to optimize the pretreatments of soil samples to realize rapid and accurate detection of soil nitrogen by NIR spectroscopy.

## Figures and Tables

**Table 1:** The PLS model prediction of different soil particle sizes.

Particle size (mm)	Pretreatments	Calibration Set		Prediction Set		
		Rc^2^	RMSEC (g/kg)	Rp^2^	RMSEP (g/kg)	RPD
1–2	Origin	0.567	0.082	0.716	0.081	1.734
	S-G	0.567	0.082	0.716	0.081	1.734
	MSC	0.685	0.065	0.900	0.048	3.002
	SNV	0.735	0.064	0.871	0.049	2.815
	1st-Der	0.713	0.064	0.893	0.050	2.938
0.45–1	Origin	0.770	0.062	0.854	0.051	2.645
	S-G	0.772	0.061	0.858	0.050	2.739
	MSC	0.912	0.036	0.837	0.056	2.429
	SNV	0.928	0.033	0.795	0.065	2.061
	1st-Der	0.947	0.029	0.786	0.068	2.082
0.28–0.45	Origin	0.849	0.050	0.866	0.048	2.699
	S-G	0.847	0.050	0.867	0.048	2.706
	MSC	0.800	0.059	0.824	0.042	2.284
	SNV	0.800	0.058	0.827	0.040	2.415
	1st-Der	0.927	0.035	0.835	0.054	2.446
0.18–0.28	Origin	0.967	0.023	0.976	0.020	6.303
	S-G	0.967	0.023	0.972	0.019	6.706
	MSC	0.949	0.019	0.968	0.021	5.036
	SNV	0.969	0.022	0.950	0.019	4.811
	1st-Der	0.970	0.023	0.969	0.021	5.706
0–0.18	Origin	0.979	0.021	0.937	0.032	3.932
	S-G	0.993	0.011	0.917	0.037	3.422
	MSC	0.959	0.026	0.929	0.036	3.717
	SNV	0.960	0.025	0.926	0.036	3.659
	1st-Der	0.964	0.023	0.891	0.047	3.066
0–2	Origin	0.926	0.026	0.839	0.051	2.468
	S-G	0.932	0.034	0.839	0.051	2.482
	MSC	0.942	0.031	0.865	0.041	2.773
	SNV	0.940	0.032	0.879	0.041	3.659
	1st-Der	0.927	0.035	0.874	0.045	3.154

**Table 2:** The PLS model prediction of different soil particle sizes.

Particle Size (mm)	Calibration Set		Prediction Set		
	Rc^2^	RMSEC (g/kg)	Rp^2^	RMSEP (g/kg)	RPD
a + b	0.732	0.064	0.741	0.072	1.969
a + c	0.516	0.088	0.634	0.083	1.616
a + d	0.353	0.011	0.286	0.113	1.142
a + e	0.160	0.118	0.101	0.121	1.042
b + c	0.600	0.081	0.737	0.072	1.858
b + d	0.344	0.105	0.487	0.098	1.332
b + e	0.140	0.118	0.243	0.124	1.065
c + d	0.415	0.099	0.577	0.092	1.410
c + e	0.674	0.075	0.637	0.077	1.638
d + e	0.750	0.064	0.852	0.056	2.295

Soil particle sizes: (a) 1–2 mm; (b) 0.45–1 mm; (c) 0.28–0.45 mm; (d) 0.18–0.28 mm; (e) 0–0.18 mm.

## Supplementary Materials

Supplementary Materials are available online, Table S1: The CARS-PLS model prediction of different soil particle sizes Table S2: The bi-PLS model prediction of different soil particle sizes. Table S3: The GA-PLS model prediction of different soil particle sizes. Table S4: The SPA-PLS model prediction of different soil particle sizes.
