# Supplementary material for: Application of Near-infrared Spectroscopy and Multiple Spectral Algorithms to Explore the Effect of Soil Particle Sizes on Soil Nitrogen Detection
Source: Molecules. 2019 Jul 7;24(13):2486. doi: 10.3390/molecules24132486 (PMC6651272; doi:10.3390/molecules24132486)
Supplement: Supplementary file 1 [file molecules-24-02486-s001.pdf]

Supplymentary materials

Application of Near-infrared Spectroscopy and Multiple Spectral Algorithms to Explore the Effect of Soil Particle Sizes on Soil Nitrogen Detection

Shupei Xiao [1,2] and Yong He [1,2,*]

[1] College of Biosystems Engineering and Food Science, Zhejiang University, Hangzhou 310058, China; 180312@zju.edu.cn

[2] Key Laboratory of Spectroscopy Sensing, Ministry of Agriculture, P. R. China

* Correspondence: yhe@zju.edu.cn; Tel.: +86-0571-8898-2143

Table S1. The CARS-PLS model prediction of different soil particle sizes.

Particle Size (mm)	Pretreatments	Calibration Set		Prediction Set		
		Rc^2	RMSEC (g/kg)	Rp^2	RMSEP (g/kg)	RPD
1–2	Origin	0.780	0.058	0.724	0.073	1.927
	S–G	0.730	0.065	0.738	0.072	1.943
	MSC	0.691	0.079	0.889	0.065	2.225
	SNV	0.709	0.065	0.839	0.055	2.497
	1st–Der	0.729	0.065	0.733	0.073	2.029
0.45–1	Origin	0.899	0.041	0.860	0.052	2.604
	S–G	0.912	0.038	0.819	0.059	2.310
	MSC	0.897	0.039	0.845	0.053	2.543
	SNV	0.923	0.034	0.810	0.062	2.178
	1st–Der	0.908	0.037	0.851	0.045	2.969
0.28–0.45	Origin	0.892	0.042	0.900	0.041	3.116
	S–G	0.918	0.037	0.944	0.034	3.767
	MSC	0.926	0.036	0.793	0.047	2.045
	SNV	0.918	0.030	0.823	0.044	2.187
	1st–Der	0.893	0.042	0.898	0.042	3.168
0.18–0.28	Origin	0.970	0.022	0.966	0.025	5.178
	S–G	0.970	0.022	0.968	0.024	5.336
	MSC	0.969	0.022	0.938	0.020	6.202
	SNV	0.968	0.023	0.950	0.019	6.840
	1st–Der	0.971	0.022	0.966	0.024	5.203
0–0.18	Origin	0.887	0.044	0.867	0.045	2.789
	S–G	0.911	0.038	0.875	0.045	2.820
	MSC	0.894	0.041	0.833	0.054	2.447
	SNV	0.896	0.041	0.824	0.054	2.412
	1st–Der	0.888	0.044	0.860	0.047	2.699
0–2	Origin	0.914	0.386	0.854	0.059	2.142
	S–G	0.890	0.043	0.857	0.057	2.229
	MSC	0.909	0.038	0.884	0.044	2.982
	SNV	0.897	0.041	0.852	0.036	3.634
	1st–Der	0.885	0.044	0.850	0.060	2.124

Table S2. The biPLS model prediction of different soil particle sizes.

Particle Size (mm)	Pretreatments	Calibration Set		Prediction Set		
		Rc^2	RMSEC (g/kg)	Rp^2	RMSEP (g/kg)	RPD
1–2	Origin	0.820	0.053	0.771	0.066	2.113
	S–G	0.826	0.052	0.782	0.065	2.165
	MSC	0.737	0.060	0.879	0.051	2.843
	SNV	0.776	0.057	0.862	0.051	2.715
	1st–Der	0.845	0.051	0.732	0.072	2.060
0.45–1	Origin	0.886	0.000	0.814	0.056	2.386
	S–G	0.889	0.042	0.853	0.052	2.638
	MSC	0.871	0.044	0.865	0.049	2.729
	SNV	0.856	0.047	0.841	0.057	2.379
	1st–Der	0.890	0.044	0.873	0.048	2.944
0.28–0.45	Origin	0.877	0.045	0.908	0.040	3.209
	S–G	0.907	0.039	0.884	0.045	2.855
	MSC	0.897	0.042	0.881	0.036	2.688
	SNV	0.878	0.046	0.876	0.037	2.603
	1st–Der	0.864	0.047	0.852	0.055	2.397
0.18–0.28	Origin	0.970	0.022	0.964	0.025	5.137
	S–G	0.971	0.022	0.966	0.024	5.203
	MSC	0.969	0.022	0.924	0.022	3.668
	SNV	0.969	0.022	0.929	0.022	3.747
	1st–Der	0.970	0.022	0.975	0.020	5.426
0–0.18	Origin	0.887	0.044	0.885	0.043	2.979
	S–G	0.897	0.041	0.804	0.059	2.160
	MSC	0.855	0.048	0.875	0.050	2.659
	SNV	0.899	0.040	0.827	0.054	2.430
	1st–Der	0.912	0.039	0.878	0.044	2.897
0–2	Origin	0.895	0.042	0.831	0.053	2.393
	S–G	0.893	0.043	0.842	0.052	2.421
	MSC	0.906	0.040	0.834	0.048	2.770
	SNV	0.894	0.042	0.843	0.055	2.377
	1st–Der	0.899	0.041	0.895	0.041	3.088

Table S3. The GA–PLS model prediction of different soil particle sizes.

Particle Size (mm)	Pretreatments	Calibration Set		Prediction Set		
		Rc^2	RMSEC (g/kg)	Rp^2	RMSEP (g/kg)	RPD
1–2	Origin	0.820	0.053	0.771	0.066	2.113
	S–G	0.826	0.052	0.782	0.065	2.165
	MSC	0.737	0.060	0.879	0.051	2.843
	SNV	0.776	0.057	0.862	0.051	2.715
	1st–Der	0.845	0.051	0.732	0.072	2.060
0.45–1	Origin	0.886	0.000	0.814	0.056	2.386
	S–G	0.889	0.042	0.853	0.052	2.638
	MSC	0.871	0.044	0.865	0.049	2.729
	SNV	0.856	0.047	0.841	0.057	2.379
	1st–Der	0.890	0.044	0.873	0.048	2.944
0.28–0.45	Origin	0.877	0.045	0.908	0.040	3.209
	S–G	0.907	0.039	0.884	0.045	2.855
	MSC	0.897	0.042	0.881	0.036	2.688
	SNV	0.878	0.046	0.876	0.037	2.603
	1st–Der	0.864	0.047	0.852	0.055	2.397
0.18–0.28	Origin	0.970	0.022	0.964	0.025	5.137
	S–G	0.971	0.022	0.966	0.024	5.203
	MSC	0.969	0.022	0.924	0.022	3.668
	SNV	0.969	0.022	0.929	0.022	3.747
	1st–Der	0.970	0.022	0.975	0.020	5.426
0–0.18	Origin	0.887	0.044	0.885	0.043	2.979
	S–G	0.897	0.041	0.804	0.059	2.160
	MSC	0.855	0.048	0.875	0.050	2.659
	SNV	0.899	0.040	0.827	0.054	2.430
	1st–Der	0.912	0.039	0.878	0.044	2.897
0–2	Origin	0.895	0.042	0.831	0.053	2.393
	S–G	0.893	0.043	0.842	0.052	2.421
	MSC	0.906	0.040	0.834	0.048	2.770
	SNV	0.894	0.042	0.843	0.055	2.377
	1st–Der	0.899	0.041	0.895	0.041	3.088

Table S4. The SPA–PLS model prediction of different soil particle sizes.

Particle Size (mm)	Pretreatments	Calibration Set		Prediction Set		
		Rc^2	RMSEC (g/kg)	Rp^2	RMSEP (g/kg)	RPD
1–2	Origin	0.753	0.060	0.754	0.071	1.970
	S–G	0.753	0.060	0.755	0.071	1.976
	MSC	0.655	0.070	0.887	0.046	3.186
	SNV	0.652	0.070	0.808	0.060	2.304
	1st–Der	0.753	0.060	0.755	0.071	2.080
0.45–1	Origin	0.852	0.087	0.879	0.050	2.677
	S–G	0.861	0.050	0.854	0.050	2.733
	MSC	0.853	0.044	0.865	0.049	2.729
	SNV	0.856	0.047	0.839	0.052	2.584
	1st–Der	0.871	0.042	0.856	0.047	3.045
0.28–0.45	Origin	0.842	0.052	0.892	0.040	3.209
	S–G	0.907	0.039	0.884	0.045	2.855
	MSC	0.799	0.051	0.906	0.029	3.255
	SNV	0.799	0.051	0.906	0.029	3.303
	1st–Der	0.842	0.052	0.892	0.040	3.286
0.18–0.28	Origin	0.947	0.029	0.962	0.022	5.710
	S–G	0.954	0.027	0.966	0.023	5.476
	MSC	0.951	0.208	0.945	0.021	3.973
	SNV	0.940	0.029	0.910	0.027	3.912
	1st–Der	0.954	0.027	0.966	0.023	4.834
0–0.18	Origin	0.861	0.049	0.904	0.038	3.332
	S–G	0.858	0.048	0.864	0.045	2.813
	MSC	0.839	0.051	0.924	0.037	3.617
	SNV	0.839	0.051	0.924	0.037	3.585
	1st–Der	0.864	0.048	0.864	0.045	2.813
0–2	Origin	0.793	0.059	0.782	0.058	2.175
	S–G	0.789	0.059	0.794	0.058	2.179
	MSC	0.810	0.055	0.791	0.057	2.331
	SNV	0.834	0.053	0.828	0.056	2.343
	1st–Der	0.843	0.049	0.834	0.050	2.522
